# Surface microhardness and depth of cure in bulk fill resin composites with and without preheating: An *in vitro* study

**DOI:** 10.4317/jced.62844

**Published:** 2025-07-01

**Authors:** Brisseth Pérez-Pachas, Fiorella Shibuya-Panduro, Leonor Castro-Ramirez, Luis Cervantes-Ganoza, Marysela Ladera-Castañeda, Jose Huamani-Echaccaya, César Cayo-Rojas

**Affiliations:** 1 Universidad Privada San Juan Bautista, School of Stomatology, Lima, Peru

## Abstract

**Background:**

Preheating bulk-fill resins reduces their viscosity, facilitates their adaptation, and improves polymerization by accelerating the activity of photoinitiators. This would allow for more efficient application and deeper curing. The study’s objective was to compare the surface microhardness and depth of cure of three bulk-fill composite resins with and without preheating.

**Material and Methods:**

Sixty samples were prepared from three different materials, Aura Bulk Fill, Filtek One Bulk Fill, and Opus Bulk Fill, in standardized molds. A Vickers electronic hardness tester was used to measure the surface microhardness. The depth of cure was calculated using the upper-to-lower microhardness ratio for each sample. Robust Student’s T was used to compare two independent measures, and Welch’s robust ANOVA was used to compare more than two independent measures with Games Howell’s post hoc. Significance was set at *p*<0.05.

**Results:**

It was observed that the preheated Aura Bulk Fill and Filtek One Bulk Fill resins had significantly higher surface microhardness than the same resins without preheating (*p*<0.001 and *p*=0.038, respectively). On the other hand, the non-preheated Opus Bulk Fill resin had significantly higher surface microhardness than the same resin preheated (*p* = 0.046). In addition, it was evident that the Filtek One Bulk Fill resin had a greater depth of cure than the Aura Bulk Fill resin (*p* = 0.011). This, in turn, had a greater depth of cure than Opus Bulk Fill resin (*p* = 0.003). Finally, with and without preheating, no significant differences existed in the depth of cure of the Bulk Fill resins evaluated (*p*>0.05).

**Conclusions:**

Preheating significantly increased the surface microhardness in Aura Bulk Fill and Filtek One Bulk Fill resin, while it caused the opposite effect in Opus Bulk Fill resin. In addition, preheating did not increase the depth of cure of each Bulk Fill resin evaluated. Finally, with and without preheating, Filtek One Bulk Fill resin presented higher surface microhardness and depth of cure than Aura Bulk Fill and Opus Bulk Fill resins.

** Key words:**Preheating, bulk-fill composite resin, depth of cure, microhardness.

## Introduction

Resin composites are the most widely used dental restorative materials. They follow the principles of minimally invasive dentistry, offer an esthetically pleasing appearance, and possess physical, chemical, and mechanical properties that resemble tooth structures (1,2).

With the evolution of these materials, bulk-fill resin composites appeared. These composites allow a monoblock technique, placing a restoration with a 4 to 5-mm thick layer, thus reducing the number of clinical steps and the shrinkage effect. In addition, their composition contains polymerization accelerators that reduce the light-curing time (3-5).

Bulk fill resins are composed similarly to traditional filler resins. However, each manufacturer adds some modifications to improve their properties, such as modified monomers, flexible fillers, or even photoinitiators to achieve proper polymerization and decrease the stress load during this process (3,6).

Although manufacturers recommend a single filler increment of 4 mm, insufficient polymerization can decrease the material’s mechanical and biological properties (7,8). Therefore, measuring the depth of cure is a crucial indicator of the level of effective polymerization achieved in bulk fill resins (7,9).

Mechanical properties are important when choosing the appropriate restorative material because they greatly affect its durability (1). Surface microhardness is one of the fundamental properties that ensure the resin’s durability. It protects the surface against damage due to compressive forces, polishing wear, or abrasive effects applied to the material (2,10).

Due to the great versatility of resin composites and their superior mechanical properties, alternative methods have been used to decrease their viscosity; the thermoplastic technique is the best known since it is with this technique that the resin becomes more fluid with increasing temperature (11,12). Preheating prior to light activation exerts a pronounced effect on the polymerization kinetics of the multifunctional methacrylate monomers that comprise the primary component of dental restorative materials (12). This thermal pretreatment enhances molecular mobility, thereby increasing the degree of conversion and reducing residual unreacted monomers (12,14). Preheating the bulk fill resins before polymerization would improve their handling by reducing the film thickness and viscosity, decreasing shrinkage stress, and reducing microleakage (15). This transient reduction in viscosity renders the material akin to a flowable composite without compromising its favorable mechanical characteristics (14,16). Moreover, preheating augments various physical and mechanical properties—such as surface hardness, flexural strength, and tensile strength—thereby enhancing restorations’ longevity and clinical performance (15,17).

Therefore, the present study aimed to compare the surface microhardness and depth of cure of three bulk-fill composite resins with and without preheating. The null hypotheses were: 1) There are no significant differences when comparing the surface microhardness of bulk fill resins with and without preheating, and 2) There are no significant differences when comparing the depth of cure of bulk fill resins with and without preheating.

## Material and Methods

1. Study design

This experimental *in vitro* study was performed between August and September 2024 at the “High Technology Laboratory Certificate” laboratory and at the School of Stomatology of the Universidad Privada San Juan Bautista (UPSJB), Lima, Peru. In addition, it considered the CRIS (Checklist for Reporting In-vitro Studies) guideline (18).

2. Ethical Considerations

This research was approved by the Institutional Research Ethics Committee of the School of Stomatology of the Universidad Privada San Juan Bautista with letter No.1404-2024-CIEI-UPSJB on August 08, 2024.

3. Sample Calculation and Selection

**Figure 1:**
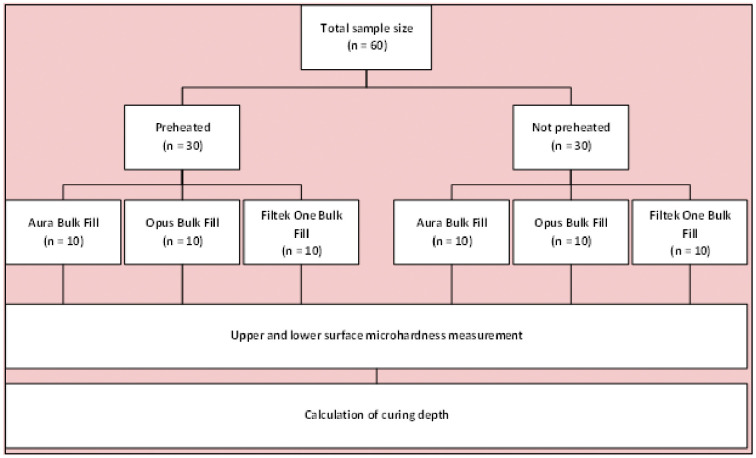
Random distribution of the samples, according to group conformation.

The total sample size was n = 60. This was a higher number than calculated based on the data obtained in a pilot study prior to the definitive experiment, where the formula was applied according to the analysis of variance in the statistical software G*Power version 3.1.9.7 considering a significance level (α) = 0.05, a statistical power (1-β) = 0.80 and an effect size 5.99 with six independent groups. Subsequently, 60 samples were manufactured and standardized, randomly distributed into two groups of 30 samples each. They were then randomly divided into three equal subgroups (n = 10) to evaluate surface microhardness and depth of cure (Fig. 1).

4. Sample preparation

Discs of Aura Bulk Fill, Filtek One Bulk Fill, and Opus Bulk Fill resin composites were prepared with a standardized mold of 6 mm diameter and 4 mm depth, which were made by a single operator (19,20) ( Table 1).

Resin composites were previously preheated with an AR Heater resin heater (Zhengzhou, Henan, China) at 70 °C for 10 min (11) and subsequently placed in a single increment in the mold. The mold was filled with resin composites, placed on a glass slide covered with a celluloid strip, and pressed to remove excess material (19,21). All samples were light-cured with an Elipar DeepCure-L LED lamp (3M, ESPE, St. Paul, MN, USA) at 1,470 mW/cm2 for 20 seconds. Subsequently, the samples were stored at 37 °C for 24 h.

5. Surface microhardness test

A Vickers electronic microhardness tester (HVS-1000 Jinan Liangong Testing Technology Co., Ltd., Shandong, China) with an approximation of 1 µ at 40X was used to perform the surface microhardness test measurement. Four indentations were made in the middle of the surface of each block under a load of 100 gf for 10 seconds at different points; each maintained a minimum distance of 1 mm from the other. The load applied to the indentation surface was divided to determine the surface microhardness (kg/mm2 = HV (Vickers hardness)). The top and bottom microhardness of each specimen was measured.

6. Measurement of the depth of cure index

**Figure 2:**
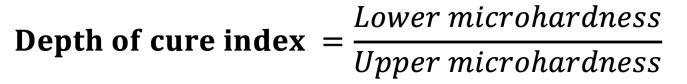
Formula.

The depth of cure index values of the resin composites were obtained by calculating each sample’s surface microhardness ratio. This ratio was calculated by dividing the average values of the top and bottom surfaces using the following equation (19,21,22), (Fig. 2).

7. Statistical analysis

The data was imported by SPSS v24.0 statistical software. For descriptive analysis, measures of central tendency and dispersion, such as mean and standard deviation, were used. For inferential analysis, after assessing normality and homoscedasticity with Shapiro Wilk and Levene’s test, respectively, robust Student’s t-test was used to compare two independent measures, and Welch’s robust ANOVA was used to compare more than two independent measures with Games Howell’s post hoc. Bar graphs at 95% confidence were used to represent the averages of three Bulk Fill resins with and without preheating. All statistical analysis was set at a significance level of *p*<0.05.

## Results

**Figure 3:**
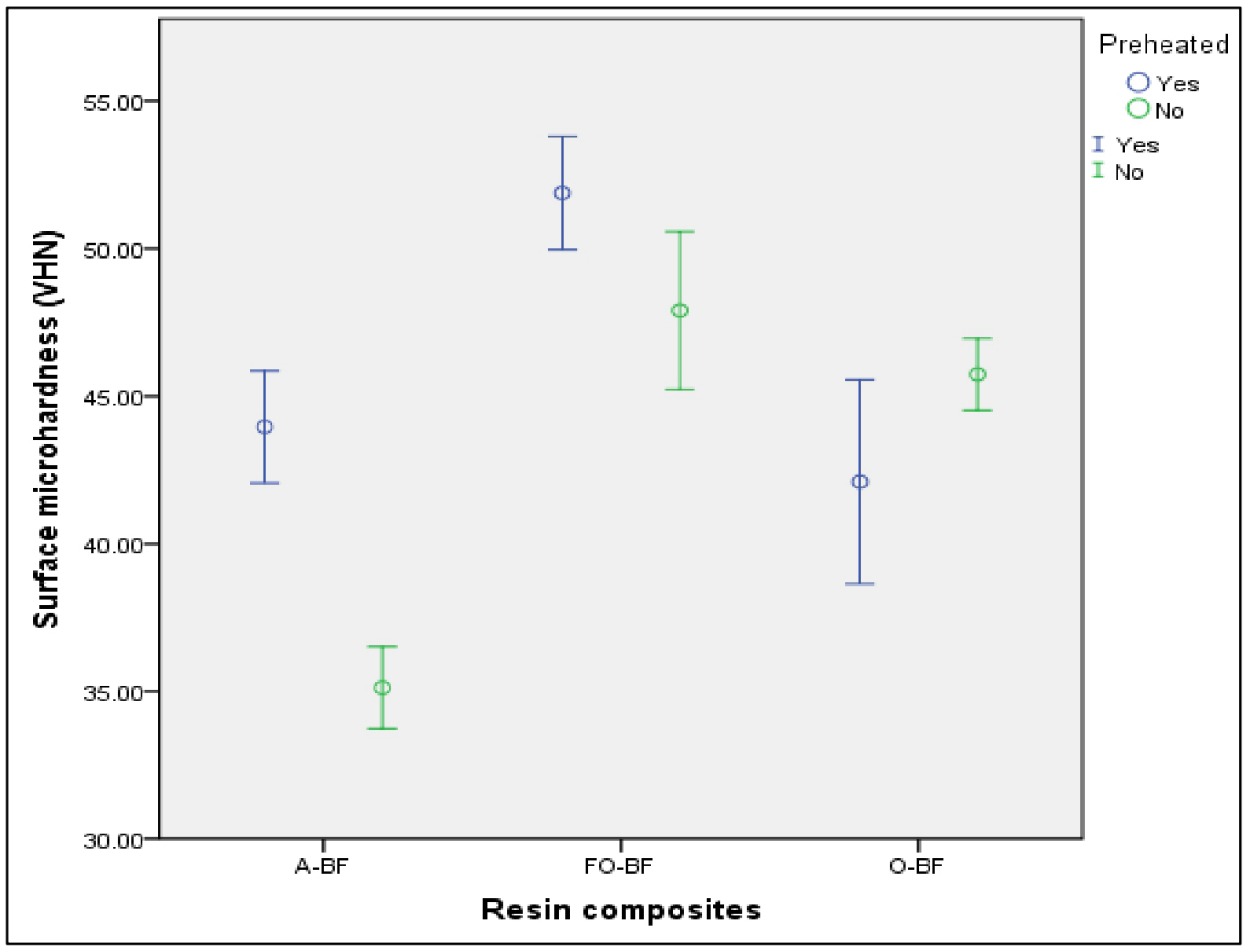
Average surface microhardness (VHN) of three bulk-fill resins, with and without preheating, at 95% confidence interval. A-BF: Aura Bulk Fill, FO-BF: Filtek One Bulk Fill, O-BF: Opus Bulk Fill.

It was observed that the preheated Aura Bulk Fill and Filtek One Bulk Fill resin composites had significantly higher surface microhardness compared to the same resins without preheating (*p*<0.001 and *p*=0.038, respectively). On the other hand, the non-preheated Opus Bulk Fill resin had significantly higher surface microhardness than the preheated resin (*p* = 0.046) ( Table 2, Fig. 3).

In addition, when comparing the three preheated resins, it was significantly observed that the Filtek One Bulk Fill resin had higher surface microhardness compared to the Aura Bulk Fill and Opus Bulk Fill resins (*p* <0.001 and *p* <0.001, respectively). No significant differences existed between the latter two resins (*p* = 0.549). On the other hand, when comparing the resins without preheating, it was observed that Filtek One Bulk Fill resin had significantly higher surface microhardness than Opus Bulk Fill resin (*p* = 0.001), and this, in turn, higher surface microhardness than Aura Bulk Fill resin (*p* <0.001) ( Table 3).

**Figure 4:**
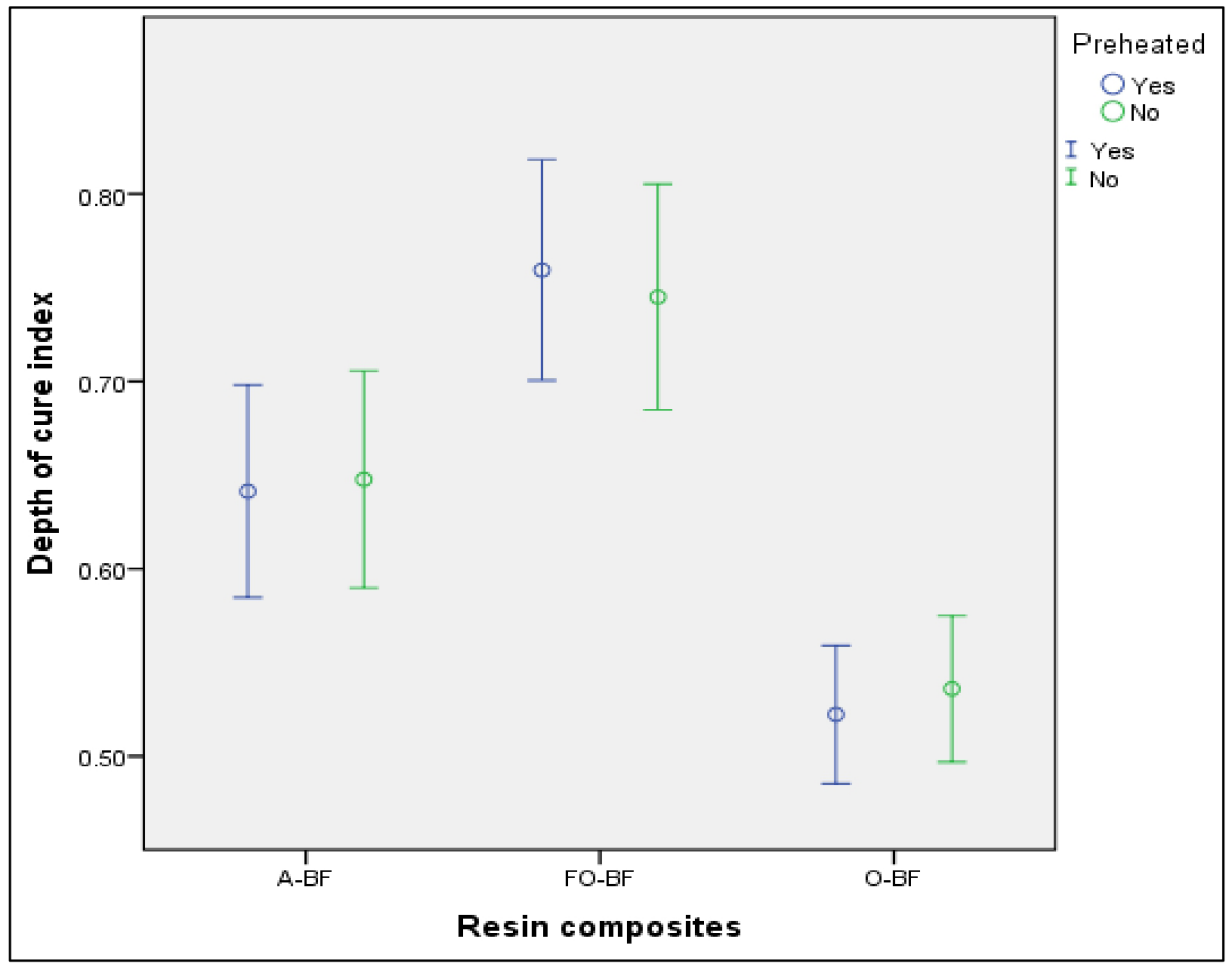
Average depth of cure index of three bulk fill resins, with and without preheating, at 95% confidence interval. A-BF: Aura Bulk Fill, FO-BF: Filtek One Bulk Fill, O-BF: Opus Bulk Fill.

It was evidenced that the preheated resins Aura Bulk Fill, Filtek One Bulk Fill, and Opus Bulk Fill presented similar depth of cure compared to the same resins without preheating (*p* = 860, *p* = 0.705 and 0.571, respectively) ( Table 4, Fig. 4).

When comparing the three preheated resins, it was significantly observed that the Filtek One Bulk Fill resin had a greater depth of cure than the Aura Bulk Fill resin (*p* = 0.011), which in turn had a greater depth of cure than the Opus Bulk Fill resin (*p* = 0.003). Likewise, when comparing the resins without preheating, it was significantly observed that the Filtek One Bulk Fill resin had a greater depth of cure than the Aura Bulk Fill resin (*p* = 0.043), and this, in turn, had a greater depth of cure than the Opus Bulk Fill resin (*p* = 0.006) ( Table 5, Fig. 4).

## Discussion

Surface microhardness and depth of cure are critical indicators to evaluate the mechanical properties and durability of bulk-fill resins (23), which could be improved by preheating the resins before polymerization (15,17). In this study, the surface microhardness and depth of cure of Filtek One bulk-fill (FO-BF), Aura bulk-fill (A-BF), and Opus bulk-fill (O-BF) resins with and without preheating were evaluated and compared. The surface microhardness (VHN) of the preheated A-BF and FO-BF resins was significantly higher than those without preheating. However, O-BF reduced its VHN value after preheating. Additionally, it was evidenced that the FO-BF resin, both with and without preheating, had a greater depth of cure than the Aura Bulk Fill and Opus Bulk Fill resins. Due to these results, the formulated null hypotheses were rejected.

The FO-BF and A-BF resins had better surface microhardness when preheated, while O-BF presented better microhardness without preheating. This was in agreement with that obtained by Keles *et al*. (24) and Degirmenci *et al*. (25), who found better properties in one group of preheated resins, while in the other group, the microhardness decreased. They also state that preheating resins mainly improve their microhardness due to decreased intermolecular forces, facilitating their clinical handling and adaptation to the dental cavity (24,26). This process increases the mobility of free radicals. It favors the frequency of collisions between the active group and the radicals, thus improving the degree of conversion during polymerization and increasing the microhardness of the bulk-fill resin (24-26). However, the different effects obtained between resin groups may be due to Degirmenci *et al*. (25), who state that the effectiveness of preheating is related to the matrix’s chemical composition and the filler’s amount. Opus Bulk Fill O-BF resin incorporates APS (Advanced Polymerization System) in its composition. It is considered an innovative alternative photoinitiator whose photoinitiation mechanism optimizes polymerization even under low light intensity conditions (27,28). Moreover, this system combines initiators and co-initiators that improve the efficiency of the degree of conversion, favoring the mechanical strength and stability of the material (27,28). Several studies have reported the same behavior in resins with alternative photoinitiators, such as ivocerin, within their composition (24,26,29). This is because the interaction of the heat applied to the resin composites would cause a differentiation in the organic matrix structure of the material and the formation of weak linear bonds instead of strong cross-links (24). In turn, the preheating temperature could have caused premature decomposition of the photoinitiators, altering the polymerization kinetics and reducing the conversion of monomers to polymer. It could also have intensified polymerization shrinkage, generating internal stresses that compromise the final structure of the material. As a result of these alterations, the surface microhardness of the O-BF resin probably decreased compared to the same resin without preheating (26,29). This leads us to believe preheating would only be effective in camphorquinone-based resin composites.

In general, thermal pretreatment can intensify polymerization shrinkage (26,29) since elevated temperatures increase monomer mobility, accelerating the reaction rate and increasing the degree of conversion during light-curing (13,14). Although this higher conversion may improve certain mechanical properties of the composite—such as surface hardness and flexural strength (15,17)—it also yields a more densely cross-linked polymer network and, consequently, greater volumetric contraction (30,31). When shrinkage occurs rapidly, it can exceed the material’s capacity to dissipate internal stresses, resulting in residual stresses (32,33) that compromise the long-term integrity of the restoration. To mitigate these effects, manufacturers have incorporated urethane-dimethacrylate (UDMA) into their resin formulations; UDMA’s intrinsic chain-relaxation ability moderates network densification during polymerization, reducing shrinkage stress (30). All three resin composites evaluated contained UDMA; however, in the case of OPUS—which utilizes an alternative photoinitiator system—thermal pretreatment adversely affected this system, altering both polymerization kinetics and the ultimate degree of conversion (26,29).

Another relevant property to evaluate in this type of resin composite is the depth of cure in increments of 4 mm or more, as indicated by the manufacturers (34). This investigation observed that the preheated FO-BF, A-BF, and O-BF resins showed a similar depth of cure to the same resins without preheating. This result agreed with that obtained by Keles *et al*. (24); this may be because when the resin composites are placed, they cool down, decreasing their temperature by 35% to 40% (25). In addition, this property also depends mainly on the characteristics of the resin composite network formation, so the light intensity and the translucency of the resin would also affect the depth of cure (35).

The preheated and non-preheated FO-BF resin obtained a higher depth of cure than the A-BF and O-BF resin; this fact may be justified by the photoinitiator camphorquinone (CQ), which is sensitive to wavelengths in the blue light range (460-480 nm) (36,37), which coincides with the emission spectrum of monowave lamps, which belongs to the type of lamp used in this study. These results agreed with those reported by Gutierrez *et al*. (38), who found that camphorquinone-based resin composites obtained better results when light-cured by monowave LEDs. By emitting a concentrated wavelength in this range, monowave lamps optimize the energy absorption by camphorquinone, ensuring an efficient and uniform activation of the polymerization reaction (36,37). In turn, A-BF exhibited a higher depth of cure than O-BF, which could be due to its ultra-high density (UHD) glass filler composition, which provides a high-strength interface that can withstand high compressive forces and can improve the polymerization efficiency (39). It was again found that the O-BF resin was the least favored resin despite having the manufacturer’s secr*et al*ternative initiators and co-initiators that amplify the polymerization capacity and increase both the degree of conversion and depth of cure (27,28), so this could be evidenced by the application of a Polywave-type LED lamp. It is worth mentioning that, of the three bulk fill resins applied at 4 mm depth, none reached a perfect indicator since the ratio between the upper and lower microhardness is expected to be 100% (1.00). However, it is acceptable for resin composites to approach or reach 80% (0.80) (24,40), with FO-BF approaching this acceptable threshold.

The importance of this study is that preheating can be effective. However, factors such as the type of light-curing unit and material composition must be taken into account. Therefore, clinicians should weigh the choice of a resin composite and the application technique, as this could compromise the surface microhardness and depth of cure, which may have detrimental effects on its longevity.

As a strength of the study, the Vickers test was used since it is the most widely used to measure surface microhardness and depth of cure, providing reliable results; in addition, they do not damage the surface of the samples, and the tests can be repeated consistently (24). Also, performing these tests together comprehensively evaluates the polymerization efficiency (24). It is worth mentioning that in this study, an accepted method was used to evaluate the depth of cure because it correlates well with the degree of conversion and surface microhardness of resin composites since it has been shown that other methods, such as optical microscopy and scratch tests, would overestimate the depth of cure (25,41).

Of the limitations, the results may not fully reflect the clinical performance of the evaluated resin composites, as the studies were conducted under *in vitro* conditions. The molds used to place the composites were at room temperature, which might differ from the temperature conditions in the oral cavity (2,11,28). In addition, not many studies related to the topic could make comparisons since the little evidence available worked with other resin composites (24,25) and, in some cases, investigated the placement of layered resin composites (40). However, the bulk fill resins used in the present study are designed to be placed in 4-mm increments.

Since the impact of preheating on the depth of polymerization may vary depending on the material, *in vitro* evaluations of the bulk fill resins are recommended before applying them in a clinical setting. In addition, it would be interesting to investigate the influence that monowave and polywave curing units may have on the resin composites evaluated. It is also recommended that other mechanical properties, such as the surface roughness of the bulk fill resins, their wear resistance, and their color stability, be evaluated. Dentists’ perception of the preheating of bulk fill resins should also be explored, as it could provide valuable information on their clinical acceptance and impact on the restorations’ handling, adhesion, and final quality. Furthermore, *in vitro* investigations, such as the one presented, are an essential first step in evaluating the properties of new dental materials; however, it is imperative to complement them with long-term randomized clinical trials to confirm their efficacy and safety under real conditions. This comprehensive approach would allow optimization of both materials and techniques used in restorative dentistry.

## Conclusions

Recognizing the limitations of the present *in vitro* study, it is concluded that preheating significantly increased the surface microhardness in Aura Bulk Fill and Filtek One Bulk Fill resin, while it caused the opposite effect in Opus Bulk Fill resin. In addition, preheating did not improve the depth of cure of each Bulk Fill resin evaluated. Finally, with and without preheating, Filtek One Bulk Fill resin had significantly higher surface microhardness and depth of cure than Aura Bulk Fill and Opus Bulk Fill resins.

## Figures and Tables

**Table 1:** Technical profile of products used.

Product	Composition	Filler % (wt-vol)	Manufacturer	Lot
Filtek One Bulk Fill Restorative A2	Matrix: AUDMA, UDMA, AFM y 1, 12-dodecane-DMA Filler: not agglomerated/not aggregated silica, not agglomerated/not aggregated zirconia, aggregated zirconia / silica compound, ytterbium trifluoride	76.5 wt% 58.5 vol%	3M, ESPE, St. Paul, MN, USA	10214054
Aura bulk Fill	Matrix: Bis-GMA, UDMA Filler: silica, barium glass	65.0 wt% 81.0 vol%	SDI, Bayswater, Victoria, Australia	1212733
Opus Bulk Fill APS A2	Matrix: bis-GMA, UDMA Filler: Nanofiller Photoinitiation -Advanced Polymerization System (APS). Inorganic load of silanized silicon dioxide (silica), barium glass aluminosilicate	76.5 wt% 58.4 vol%	FGM, Santa Catarina, Brazil	250324 230224

**Table 2:** Descriptive values and comparison of the surface microhardness (VHN) of three Bulk Fill resin composites with and without preheating.

Resin composite	Preheated	n	Mean	SD	95% CI		p*	p**
					LL	UL		
A-BF	Yes	10	43.96	2.65	42.07	45.85	0.950	<0.001**
	No	10	35.12	1.93	33.74	36.50	0.319	
FO-BF	Yes	10	51.88	2.69	49.96	53.80	0.110	0.038**
	No	10	49.53	1.90	48.17	50.89	0.779	
O-BF	Yes	10	42.10	4.84	38.64	45.56	0.096	0.046**
	No	10	45.74	1.71	44.52	46.96	0.184	

A-BF: Aura Bulk Fill, FO-BF: Filtek One Bulk Fill, O-BF: Opus Bulk Fill; n: sample size, SD: Standard Deviation; 95% CI: 95% Confidence Interval; LI: Lower Limit, UL: Upper Limit. *Based on Shapiro Wilk’s normality test (*p* >0.05, normal distribution). **Based on Student’s robust t-test (*p*p* <0.05, significant differences).

**Table 3:** Post hoc comparison of surface microhardness (VHN) with and without preheating between three Bulk Fill resin composites.

Preheated	Resin composite	Resin composite	
		FO-BF	O-BF
Yes	A-BF	*p <0.001	p = 0.549
	FO-BF		*p <0.001
No	A-BF	*p <0.001	*p <0.001
	FO-BF		*p = 0.001

*Based on Games Howell post hoc (*p* <0.05, significant differences), after testing for significant differences (*p* <0.05) with Welch’s robust Anova test.

**Table 4:** Descriptive values and comparison of the depth of cure index of three Bulk Fill resin composites with and without preheating.

Resin composite	Preheated	n	Mean	SD	95% CI		p*	p**
					LL	UL		
A-BF	Yes	10	0.64	0.08	0.58	0.70	0.805	0.860
	No	10	0.65	0.08	0.59	0.71	0.804	
FO-BF	Yes	10	0.76	0.08	0.70	0.82	0.429	0.705
	No	10	0.74	0.08	0.68	0.81	0.306	
O-BF	Yes	10	0.52	0.05	0.49	0.56	0.334	0.571
	No	10	0.54	0.05	0.50	0.58	0.713	

A-BF: Aura Bulk Fill, FO-BF: Filtek One Bulk Fill, O-BF: Opus Bulk Fill; n: sample size, SD: Standard Deviation; 95% CI: 95% Confidence Interval; LI: Lower Limit, UL: Upper Limit. *Based on Shapiro Wilk’s normality test (*p* >0.05, normal distribution). **Based on Student’s robust t-test (*p <0.05, significant differences).

**Table 5:** Post hoc comparison of depth of cure index with and without preheating between three Bulk Fill resin composites.

Preheated	Resin composite	Resin composite	
		FO-BF	O-BF
Si	A-BF	*p = 0.011	*p = 0.003
	FO-BF		*p <0.001
No	A-BF	*p = 0.043	*p = 0.006
	FO-BF		*p <0.001

*Based on Games Howell post hoc (*p* <0.05, significant differences), after testing for significant differences (*p* <0.05) with Welch’s robust Anova test.

## Data Availability

The datasets used and/or analyzed during the current study are available from the corresponding author.
